# Bibliographical Notices

**Published:** 1855-09

**Authors:** 


*Clinical Lectures on Paralysis, Disease of the Brain, and other Affections of the Nervous System.* By ROBERT BENTLEY TODD, M. D., F. R. S. Philadelphia: Lindsay & Blakiston. 1855.

Dr. Todd's lectures contain a very full and instructive exposition of the etiology, pathology and treatment of the different forms of paralysis which presented themselves at King's College Hospital during a term of ten years. Although the work cannot be considered a systematic treatise on the subject, so much practical information is contained in its pages, and the cases are so admirably described, both as regards the symptoms of the patient and the various morbid alterations found post-mortem, that it cannot but prove a valuable and useful work to any one who studies it.

The author evidently thinks for himself, his extensive physiological and anatomical knowledge, guided by a clear and sound judgment, giving to his opinion a weight and authority second to no other living writer. In the twenty lectures which comprise the volume, eighty-seven cases are detailed, each one of them illustrative of some peculiar form of paralysis.

Not having space for extracts, we must content ourselves by saying that we consider Dr. Todd's work a most able contribution on a subject upon which more information was much to be desired.

Messrs. Lindsay and Blakiston deserve the thanks of the profession for the very handsome style in which the work is published.

---

*History of the American Medical Association, from its Organization up to January*, 1855. By N. S. DAVIS, M. D., Professor of Principles and Practice of Medicine and Clinical Medicine in Rush Medical College, &c. *To which is appended, Biographical Notices, with Portraits of the Presidents of the Association, and of the Author.* Edited by S. W. BUTLER, M. D. Philadelphia: Lippincott, Grambo & Co. 1855.

The above volume comprises a series of articles published anonymously in the New Jersey Medical Reporter, their paternity only being acknowledged when it was determined to republish them in their present more durable form. "I hope this explanation," says the author in his Preface, "will afford a sufficient apology for the freedom with which my own name is used throughout the work. The whole of it has been written in the midst of the most arduous and pressing professional duties, and without the possibility of commanding time for a revision."

Dr. Davis hasexecuted his task very satisfactorily. The history of the Association from its origin, the indifference, distrust and open opposition which retarded its organization, and its final, triumphant success, so fully and ably described by him, cannot but be perused with interest and advantage by every lover of our profession.

We sincerely hope the work will have a large circulation.

